# A Case of Pulmonary Basaloid Squamous Cell Carcinoma in a Non‐Smoking Female: Case Report and Literature Review

**DOI:** 10.1111/1759-7714.70106

**Published:** 2025-06-14

**Authors:** Tsukasa Satoh, Watanabe Hiromu, Nakamura Kimie, Mitsui Izumi, Nishikawa Keiichi, Chiba Shigehiro, Kenzo Soejima

**Affiliations:** ^1^ Department of Respiratory Medicine Yamanashi Kosei Hospital Yamanashi City Japan; ^2^ Department of Pathology Yamanashi Kosei Hospital Yamanashi City Japan; ^3^ Department of Respiratory Medicine Graduate School of Medicine University of Yamanashi Yamanashi City Japan

**Keywords:** mutation, postoperative care, squamous cell carcinoma

## Abstract

Pulmonary basaloid squamous cell carcinoma (BSCC) is a rare, high‐grade subtype of lung squamous cell carcinoma. It predominantly affects elderly male smokers and is usually diagnosed at an advanced stage. Here, we report an early‐stage BSCC in a 67‐year‐old non‐smoking female identified during routine health screening. Chest CT revealed a 30‐mm mass in the right lower lobe. PET‐CT showed mild FDG uptake without lymph node or distant metastasis. Bronchoscopy confirmed malignancy, and thoracoscopic lobectomy with mediastinal lymph node dissection was performed. Gross pathology demonstrated a polypoid tumor protruding into the bronchial lumen. Histologically, the tumor exhibited solid basaloid nests with peripheral palisading and a high nuclear‐to‐cytoplasmic ratio. Immunohistochemistry showed strong p63 and CK5/6 positivity, with weak focal p40 expression in less than 20% of tumor cells. Ki‐67 labeling index was approximately 50%. Neuroendocrine and breast cancer markers were negative. Mosaic p53 positivity was observed, and no actionable mutations were identified via next‐generation sequencing. The final diagnosis was primary pulmonary BSCC (pT1cN0M0, Stage IA3). The patient remains recurrence‐free 18 months postoperatively without adjuvant therapy. A literature review of 11 representative reports revealed BSCC typically presents in older male smokers at later stages and carries a poor prognosis. This case highlights the potential for BSCC to occur in non‐smokers and at early stages, emphasizing the importance of a multidisciplinary diagnostic approach integrating histology, immunohistochemistry, and molecular data.

## Graphical abstract

We report a rare case of early‐stage pulmonary basaloid squamous cell carcinoma (BSCC) in a non‐smoking female. The tumor was successfully treated with complete surgical resection, and the patient remains recurrence‐free after 18 months. This case highlights the potential for favorable outcomes in early‐detected BSCC among non‐smokers.

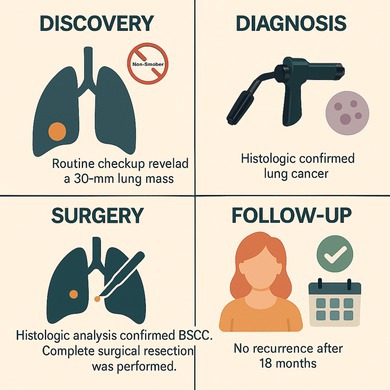

## 1 Introduction

Pulmonary basaloid squamous cell carcinoma (BSCC) is a rare histologic subtype of squamous cell carcinoma (SCC) of the lung. First described by Brambilla et al. in 1992, it is now classified in the fifth edition of the WHO classification as a high‐grade variant of SCC with poor prognosis [1]. Histologically, BSCC is defined by small basaloid cells with hyperchromatic nuclei and scant cytoplasm, forming solid nests with peripheral palisading and frequent mitotic figures.

Pulmonary BSCC typically arises in elderly male smokers and is often diagnosed at an advanced stage [2, 3]. However, rare cases have been documented in non‐smokers. The clinical and molecular characteristics, as well as the prognostic implications of BSCC in non‐smokers, remain poorly understood. We present a case of early‐stage BSCC in a non‐smoking female and examine its clinical significance in the context of the literature.

## 2 Case

**FIGURE 1:**
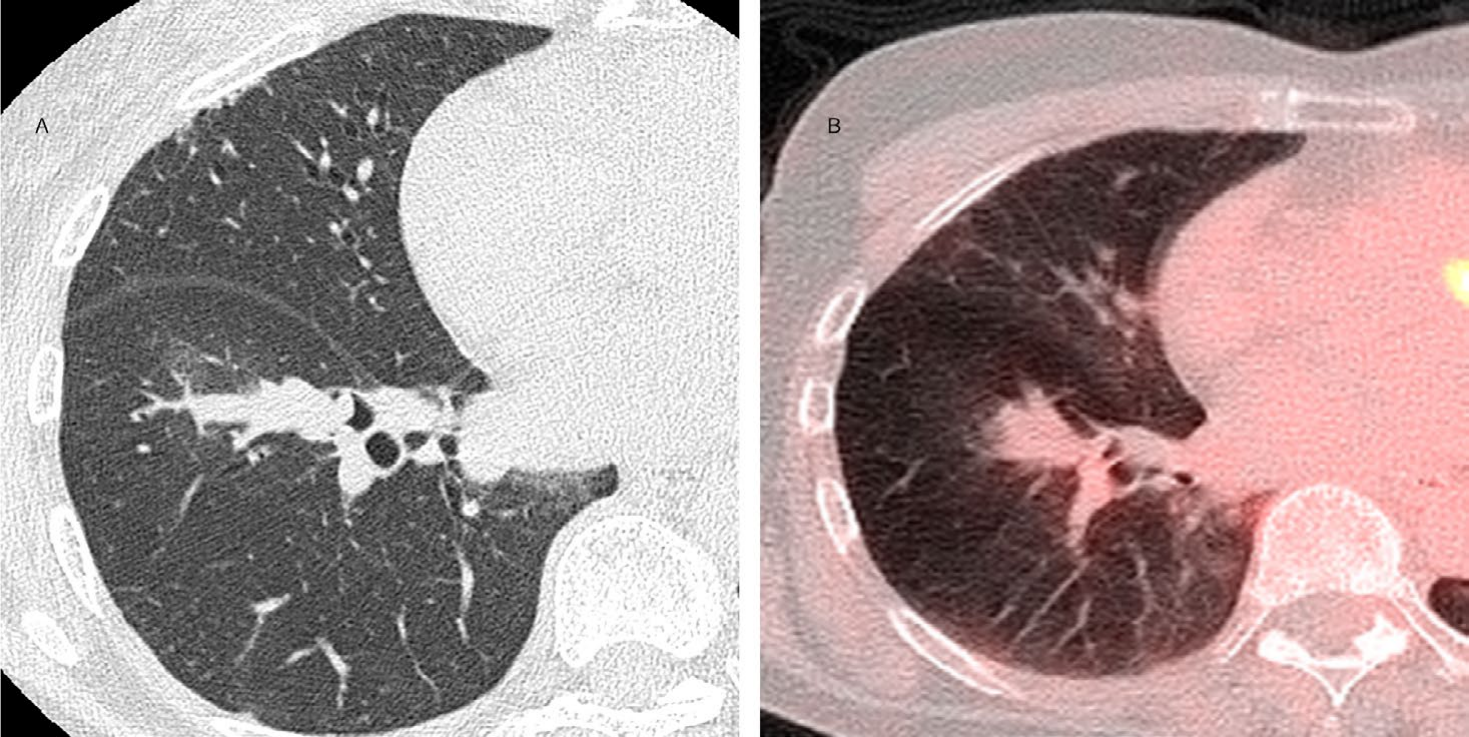
Chest imaging at the initial assessment. Computed tomography (CT) of the chest demonstrated a well‐defined 30‐mm mass in the B8/9 segment of the right lower lobe, extending into the bronchial lumen. The lesion exhibited no evidence of cavitation or necrosis. There were no signs of regional lymphadenopathy or distant metastasis. Fluorodeoxyglucose positron emission tomography‐computed tomography (FDG/PET‐CT) revealed an increased FDG uptake in the mass (B) with a maximum standardized uptake value (SUV) of 1.77. No other FDG uptake was observed.

A 67‐year‐old non‐smoking and non‐secondhand smoking woman was referred to our hospital after a 30‐mm mass was identified in the right lower lobe (B8/9 segment) on chest computed tomography during a routine health screening (Figure 1A). Her medical history included breast cancer treated by partial mastectomy 10 years earlier, with no recurrence. She had been employed as an office clerk and denied any occupational exposure to known pulmonary carcinogens. Physical examination and serum tumor markers were unremarkable. Positron emission tomography–CT showed mild fluorodeoxyglucose uptake (SUVmax 1.77) at the lesion, with no lymph node or distant metastases (Figure 1B).

**FIGURE 2:**
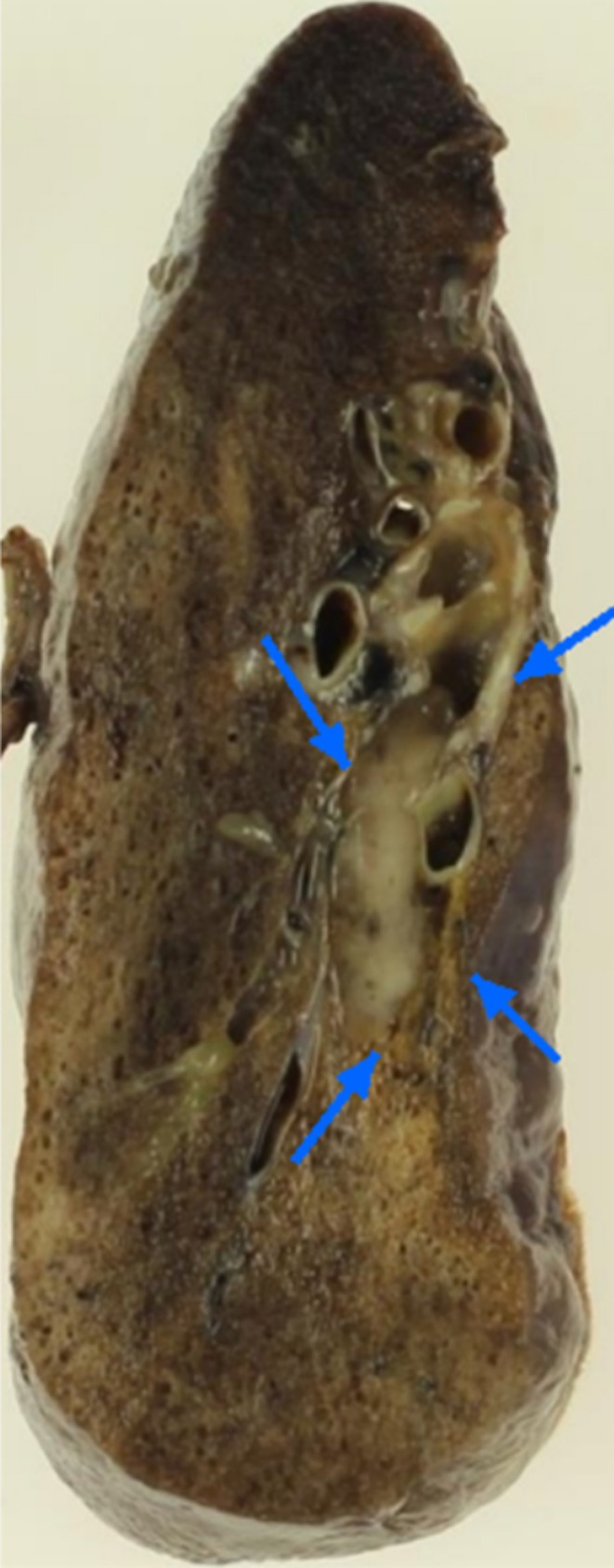
Macroscopic findings of the tumor. The tumor measured 25 × 8 mm and exhibited a polypoid growth into the bronchial lumen.

Bronchoscopy revealed a malignancy, prompting thoracoscopic right lower lobectomy and mediastinal lymph node dissection. Gross examination showed a 25 × 8 mm polypoid mass protruding into the bronchial lumen (Figure 2). No nodal metastases were identified, and the surgical margins were negative.

**FIGURE 3:**
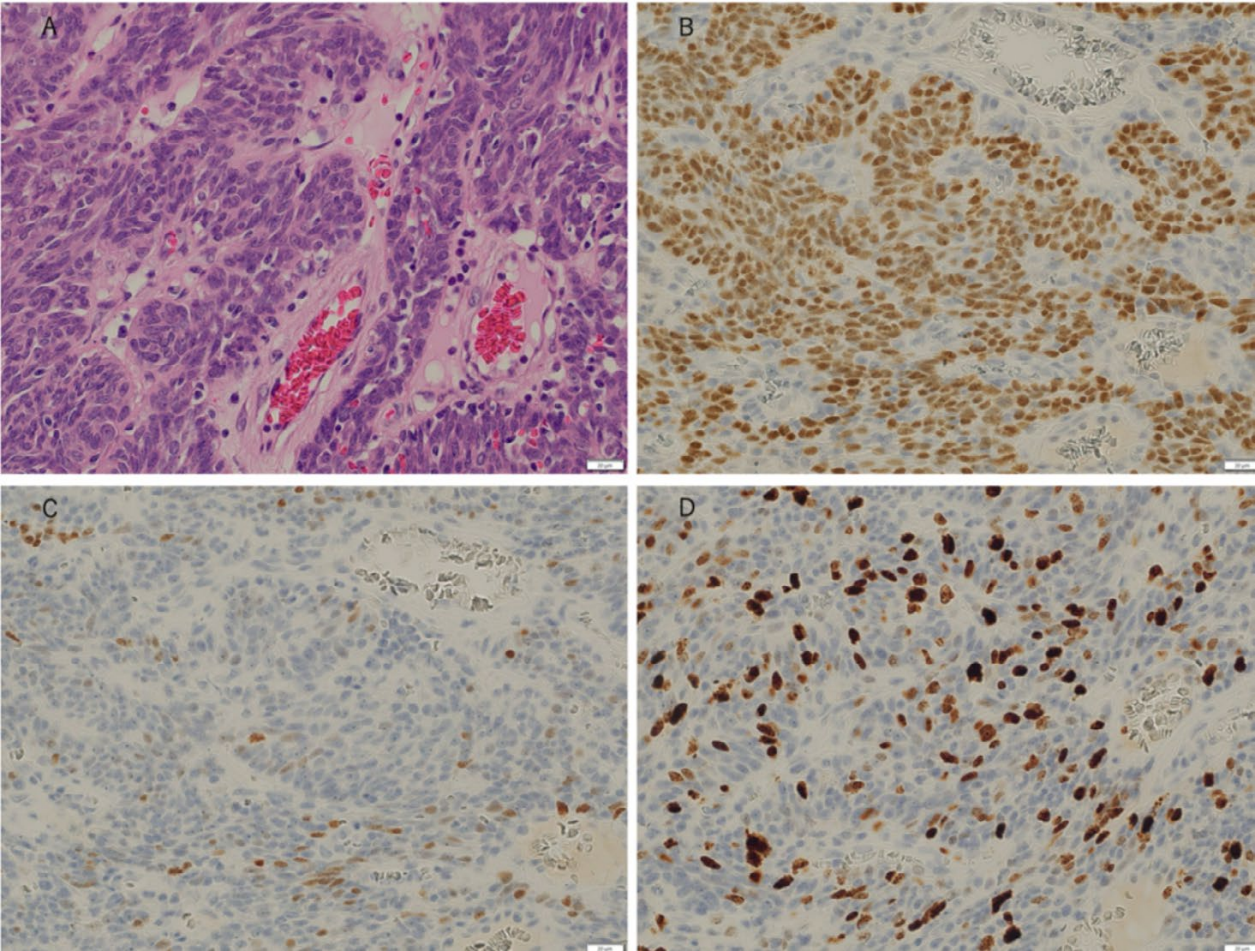
Histopathological and immunohistochemical findings of the tumor. Hematoxylin and eosin (HE) staining (A, ×400) revealed a proliferation of spindle‐shaped tumor cells arranged in a characteristic nested growth pattern with fibrous stroma. Nuclear hyperchromasia was not prominent, but mitotic figures were occasionally observed. Immunohistochemical analysis demonstrated positive staining for p63 (B, ×400). p40 (C, ×400) exhibited weak positivity. The Ki‐67 labeling index (D, ×400) was elevated at 50%, indicating a high proliferative capacity.

Histologically, the tumor was composed of densely packed basaloid cells forming solid nests within fibrous stroma, accompanied by peripheral palisading (Figure 3A). The nuclear‐to‐cytoplasmic ratio was high, with numerous mitotic figures. Immunohistochemical staining demonstrated strong nuclear positivity for p63 (Figure 3B), diffuse CK5/6 expression, and weak focal positivity for p40 in fewer than 20% of tumor cells (Figure 3C). The Ki‐67 proliferation index was approximately 50% (Figure 3D). Neuroendocrine markers (synaptophysin and chromogranin A), breast cancer markers (ER, PgR, GCDFP‐15, GATA3) and thyroid transcription factor‐1 (TTF‐1) were negative. Mosaic p53 positivity was observed. Next‐generation sequencing detected no actionable mutations. The final diagnosis was primary pulmonary BSCC (pT1cN0M0, Stage IA3). The patient remains free of disease 18 months after surgery without adjuvant treatment.

## 3 Discussion

BSCC displays distinct histological features from conventional SCC, characterized by solid nesting, increased mitotic activity, necrosis, and minimal keratinization. A definitive diagnosis requires distinction from small‐cell carcinoma and NUT carcinoma, necessitating integration of histologic, immunohistochemical, and clinical findings [4].

To provide clinical context, we reviewed prior reports of pulmonary BSCC in the literature. Table 1 summarizes the clinical and pathological characteristics of 11 representative reports, including our own.

**TABLE 1:** Clinical and pathological characteristics of reported cases of pulmonary basaloid squamous cell carcinoma.

No.	Author (year)	No. of patients (M/F)	Median or mean age (years)	Smoking history (Y/N)	Tumor size (mm)	Stage distribution (I/II/III/IV)	Immunohistochemistry profile	Genetic alterations	Outcome
1	Brambilla (1992)	Not available	Not available	Majority Yes	Not available	Stage III–IV	p63 positive, CK5/6 positive	Not available	5‐year survival rate: 15% (Stage I–II)
2	Moro (2008)	44 (43/1)	Mean 63.6 (range 37–82)	Majority Yes	> 30	I: 38, II: 28, III: 16, IV: 8	p63 positive, CK5/6 positive	Not available	5‐year survival rate: < 20% (Stage I–II)
3	Moro (1994)	37 (37/0)	Median 60.9 (range 37–79)	Yes	Not available	I: 15, II: 15, III: 6, IV: 1	Not available	Not available	5‐year survival rate: 15% (Stage I–II)
4	Yuan (2019)	425 (257/168)	Median 70.15	Not available	Not available	I: 178, II: 63, III: 101, IV: 82	Not available	Not available	5‐year overall survival: ~17.6% (all stages)
5	Miyazaki (2013)	1 (1/0)	Not available	No	28 × 23	Stage IIIA	34βE12+, CD56 (focal)+, Syn−, Chromo−, TTF‐1−	Not available	Died at 23 months
6	Fujinaga (2015)	9 (8/1)	Not available	9/0	20–50	Stage IA–IIA	34βE12+, p63+ (7/9), CK5/6+ (5/9), p40+ (3/9)	Not available	5/9 recurrence; 1 RFS 91.3 months
7	Matsuoka (2022)	3 (3/0)	Not available	3/0	19–38	Stage IA2–IB	p63+, CK5/6+, 34βE12+, p40+ (1/3)	Not available	2 alive (21, 68 months 1 died (6 months, IP)
8	Qian (2020)	13 (12/1)	Not available	10/3	Not available	Stage I–III	Not available	TP53, CDKN2A, NOTCH1	Not available
9	Wang (2011)	22 (14/8)	Mean 58.6	18/4	Mean 44	I: 9, II: 8, III: 4, IV: 1	Not available	Not available	Median OS: 19 months
10	Yamada (2019)	1 (1/0)	Not available	Yes	25 (invasive 11)	Stage IA2	p63+, CK5/6+, TTF‐1−, Syn−, Chromo−, Ki‐67: 33%	Not available	RFS > 5 years
11	Present case	1 (0/1)	67	0/1	30	Stage IA3	p63+, CK5/6+, p40 (weak+ < 20%), Ki‐67: 50%, p53 mosaic+	Not available	RFS 18 months

*Note:* Summary of clinical and pathological features of 11 published studies, including the present case, of pulmonary basaloid squamous cell carcinoma (BSCC). Data include number of patients, age, sex distribution, smoking history, tumor size, pathological stage, immunohistochemical profiles, genetic alterations, and clinical outcomes. When available, the distribution of patients across tumor stages (I–IV) and median or mean age are reported. Immunohistochemistry (IHC) profiles are presented as reported in each original study. The present case is included as Case 11.

In our patient, immunohistochemical negativity for neuroendocrine, breast cancer markers, and TTF‐1, together with the absence of clinical evidence of recurrence, supported the diagnosis of primary pulmonary BSCC [5]. BSCC typically shows p63 and CK5/6 positivity, while p40, a highly specific marker for squamous differentiation, may be weak or even negative in some cases [6, 7]. The tumor cells exhibited spindle‐shaped nuclei, distinguishing them from the round nuclei typically seen in NUT carcinoma [8]. Despite weak p40 expression, a diagnosis of BSCC was supported by morphology and immunophenotype. A definitive histologic diagnosis requires an integrated assessment of morphology, immunohistochemistry, and clinical context.

BSCC is thought to be associated with smoking and TP53 mutations [9]. In this non‐smoking patient, next‐generation sequencing revealed no targetable alterations; however, mosaic p53 positivity raised suspicion of a TP53 mutation. These findings suggest that TP53 may also play a role in the pathogenesis of BSCC in non‐smokers.

BSCC frequently occurs in elderly male smokers and is usually diagnosed at an advanced stage, with tumors often exceeding 30 mm in diameter [2, 10]. In contrast, the current case involved a non‐smoking female with a 30‐mm tumor diagnosed at Stage IA3. Although early‐stage BSCC has been associated with a high recurrence rate and a 5‐year survival rate below 20% [2], rare cases of long‐term survival have been reported, even among smokers [11]. In our case, the combination of non‐smoking status and early, complete resection may contribute to the favorable outcome. Continued surveillance remains warranted.

## 4 Conclusion

We report a case of early‐stage pulmonary BSCC in a non‐smoking female, who has remained recurrence‐free 18 months postoperatively. This case underscores that pulmonary BSCC can develop in non‐smokers and highlights the importance of comprehensive diagnosis incorporating histologic morphology, immunoprofile, and molecular data. Improved recognition and characterization of BSCC may facilitate more accurate classification and inform treatment strategies.

## Author Contributions

**Tsukasa Satoh:** drafted the manuscript and collected the data. **Watanabe Hiromu**, **Nakamura Kimie**, **Mitsui Izumi**, **Nishikawa Keiichi**, and **Chiba Shigehiro:** provided clinical expertise and revised the manuscript. **Kenzo Soejima:** supervised the project and approved the final version of the manuscript.

## Ethics Statement

The authors declare that appropriate written informed consent was obtained for the publication of this manuscript and accompanying images.

## Consent

A written informed consent was obtained from the patient to secure permission to publish the clinical history.

## Conflicts of Interest

The authors declare no conflicts of interest.

## Data Availability

The data that support the findings of this study are available from the corresponding author upon reasonable request.
